# Unveiling the multifaceted role of toll-like receptors in immunity of aquatic animals: pioneering strategies for disease management

**DOI:** 10.3389/fimmu.2024.1378111

**Published:** 2024-10-17

**Authors:** Muhammad Usman Ghani, Junfan Chen, Zahra Khosravi, Qishu Wu, Yujie Liu, Jingjie Zhou, Liping Zhong, Hongjuan Cui

**Affiliations:** ^1^ Medical Research Institute, Southwest University, Chongqing, China; ^2^ State Key Laboratory of Resource Insects, Southwest University, Chongqing, China; ^3^ State Key Laboratory of Targeting Oncology, Guangxi Medical University, Nanning, China

**Keywords:** toll-like receptors, PPR, PAMPS, innate immunity, signaling pathways, disease management

## Abstract

The pattern recognition receptor (PRR), which drives innate immunity, shields the host against invasive pathogens. Fish and other aquatic species with poorly developed adaptive immunity mostly rely on their innate immunity, regulated by PRRs such as inherited-encoded toll-like receptors (TLRs). The discovery of 21 unique TLR variations in various aquatic animals over the past several years has sparked interest in using TLRs to improve aquatic animal’s immune response and disease resistance. This comprehensive review provides an overview of the latest investigations on the various characteristics of TLRs in aquatic animals. It emphasizes their categorization, insights into 3D architecture, ligand recognition, signaling pathways, TLRs mediated immune responses under biotic and abiotic stressors, and expression variations during several developmental stages. It also highlights the differences among aquatic animals’ TLRs and their mammal counterparts, which signifies the unique roles that TLRs play in aquatic animal’s immune systems. This article summarizes current aquaculture research to enhance our understanding of fish immune systems for effective aquaculture -related disease management.

## 1 Introduction

The vertebrate immune system is an essential network for survival and adaptability since it has developed over millions of years to combat various diseases. It is composed of both adaptive and innate components. The adaptive system offers long-term immunity by retaining memory of past infections, while the innate system offers rapid defense. Identification of pathogen-associated molecular patterns (PAMPs) and priming the immune responses depend on pattern recognition receptors (PRRs), which are fundamental elements of the innate immune response (1, 2). Three families of receptors, namely Toll-like receptors (TLRs), nucleotide-binding oligomerization domain (NOD)-like receptors (NLRs), and retinoic acid-inducible gene-I (RIG)-like receptors (RLRs), are primarily involved in this process (3). TLRs were the first to be discovered among them and have drawn much interest due to their essential role in inducing innate and adaptive immunity. TLRs largely contribute to the innate immune system by initiating signaling pathways that produce cytokines and numerous inflammatory mediators upon detecting PAMPs at the site of infection (4). Over the years, these proteins have drawn more attention from the scientific community due to their vital function as early determinants of immune activation. They are thought to be present in different numbers in different species. Humans and cattle are believed to possess ten TLRs, mice have twelve, and purple sea urchins have two hundred and twenty-one TLRs (5, 6).

In aquaculture, aquatic animals are an essential source of food for humans. They encounter several challenges, including pollution, habitat degradation, and rapid variations in water temperature, resulting in disease susceptibility (7–9). In this case, TLRs are critical components of the immune system for adequate protection (10). The TLRs in aquatic animals, along with components of their signaling pathway exhibit notable structural similarities to those in the mammalian TLR system. However, aquatic animal’s TLRs also display highly distinctive features and considerable diversity, which may originate from their diverse evolutionary backgrounds and the specific ecological niches they inhabit (11). Each species of aquatic animals has varying numbers and types of TLRs, which are thought to enhance their survival by providing a wider range of pathogen recognition capabilities and enabling them to adapt to diverse environments and microbial ecologies. Comprehending the role of TLR in aquatic animal’s immunology is crucial for managing and preventing illness in aquaculture (12, 13).

Our review article aims to thoroughly investigate TLRs in species of significant economic importance that have previously been overlooked. The selection criteria for these species also consider the availability of data and their relevance to aquaculture, fisheries, and biomedical research. Additionally, by contrasting aquatic animal’s TLRs with mammals, we will be able to advance our consideration of the diverse characteristics of these receptors in various physiological contexts, from routine immune surveillance to active defense against diverse pathogens. The review also delves into the prospective avenues for long-term management plans in mariculture, which is important in food production and socioeconomic welfare.

## 2 Structural characteristics, classification, evolutionary linages and nomenclature of TLRs

### 2.1 Structural characteristics of toll-like receptors in aquatic animals

**Figure 1:**
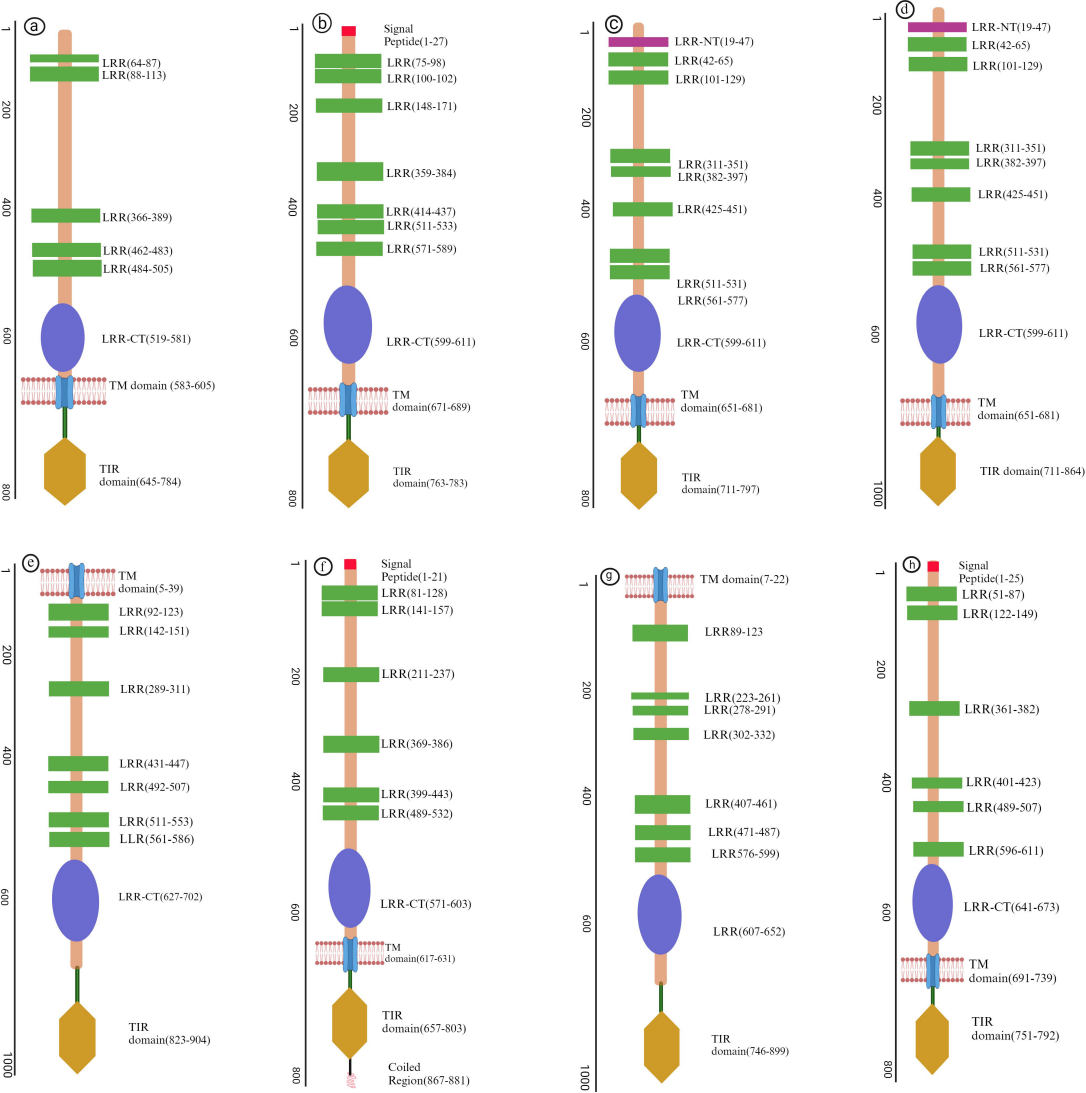
Illustrative diagrams representing the structural composition of different (TLRs) in fish. **(A)** LrTLR1; **(B)** LrTLR2; **(C)** CcTLR9; **(D)** ToTLR23; **(E)** ToTLR27; **(F)** Tr LR22; **(G)** TrTLR26; **(H)** IpTLR27. The SMART program has been used to define the structural characteristics of (TLRs) in fish, revealing a three-part domain arrangement. The figure highlights the homogeneity of fish TLR domain topologies and offers a complete perspective on their structural characteristics. (Illustrations created with BioRender.com *).* Lr, Labeo rohita; Cc, Cyprinus carpio; To, Traachinotus ovatus; Tr, Takifugu rubripes; Ip, Ictalurus punctatus.

The structural difference between TLRs relies in their structural configuration, signal transduction pathways, ligand specificity, and subcellular localization (14). TLRs are categorized into two types based on their cellular localization. Cell membrane TLRs, found on the cell’s surface, are TLRs2-1, TLRs 2-6, TLRs4, TLRs5, and TLRs10. Nucleic acid sensing or Intracellular TLRs are confined to endosomes, endoplasmic reticulum, and lysosomes, including TLRs3, TLRs7, TLRs8, and TLRs9 (15). Aquatic animal’s TLRs predominantly possess type I transmembrane proteins comprising approximately 30 signal peptides, later cleaved to produce mature protein. We utilized (the SMART) tool to predict the structural features of several TLRs in aquatic animals, such as the region of Leucine-rich repeats (LLRs) in domains of N-terminal domain (NTD) and Toll/Interleukin-1 receptor (TIR) and signal peptide presented in **Figure 1**. This comprises an extracellular domain (ECD), a transmembrane (TM) domain that makes a single membrane span, and cytosolic Toll/TIR domain; each TLR representation has an associated scale that shows the number of amino acids. Leucine-rich repeats (LRRs), essential for ligand identification and attachment, define the ECD. The TIR domain is essential for starting further signaling pathways, whereas the TM domain embeds the TLR in the cellular membrane.

#### 2.1.1 Extracellular or N-terminal domain

This domain is localized extracellularly, comprising 19–25 amino acids of Leucine-rich repeats and cysteine flanking regions that form horseshoe-shaped structures responsible for recognizing PAMPs (16, 17). Leucine-rich repeats comprise 20-30 hydrophobic amino acids with leucine-rich motif-specific sequences. TLRs in fish are architecturally similar to human TLRs and possess significant sequence similarity in their LRRs interrelating with PAMPs (18). The Leucine-rich repeats number fluctuates among fish species and TLR types. For instance, zebrafish TLR3 contains 24 LRRs, the TLR3 in the Indian carp rohu is comprised of 27 LRRs. Additionally, zebrafish TLR22 and Rohu TLR2 are made 26 and 22 LRRs respectively (19). In aquatic animals TLRs, the glycosylation pattern varies in Leucine-rich repeats that contribute pockets formation, which is crucial for PAMPs or ligands. In addition, N-terminal domains contain carbohydrate or sugar components necessary for receptors to bind pathogen-associated ligands. Notably, Toll/interleukin-1 in TLRs represent more conservation than LRRs.

#### 2.1.2 C-terminal domain

A cytoplasmic terminal domain typically refers to the intracellular portion of TLR, which is critical for downstream signaling for immune response activation (20). Regarding the functional role of secondary structure, homotypic TIR-TIR interactions are mediated by both α and β units, and the TIR motifs are extensively conserved across species in contrast to LRR motifs (21). Notably, TIR domain homologs have also been found in several disease-resistant plants. It suggesting that it was an ancient motif with immunological properties before separating plants and animals’ kingdoms.

#### 2.1.3 TM domain

Transmembrane domains are important structural components of TLRs, which are essential for innate immune response. It comprises 20 unchanged hydrophobic single-pass helix of amino acids. TMD acts as a bridge between the extracellular domain (ECD) and the cytoplasmic Toll/Interleukin-1 receptor (TIR) (22). The transmembrane domain is more common in aquatic animals and humans. Primarily, it involve in positioning of TLRs to the cell membrane, which is essential for positioning the receptor for interactions with PAMPs encountered at the cell surface or within endosomes (23). Additionally, the TMD maintains the proper alignment and spacing of the receptor within the membrane, ensuring that the ligand-binding domains are correctly presented to the extracellular environment or to endosomal contents. This spatial arrangement is vital for effective immune signaling. Furthermore, the TMDs facilitates signal transduction by linking the extracellular and intracellular signaling domains. Upon ligand binding, this connection allows conformational changes to propagate through the TMD to the intracellular domains, triggering downstream signaling pathways that orchestrate the immune response (24). The transmembrane domain is more common in - aquatic animals and humans.

**Figure 2:**
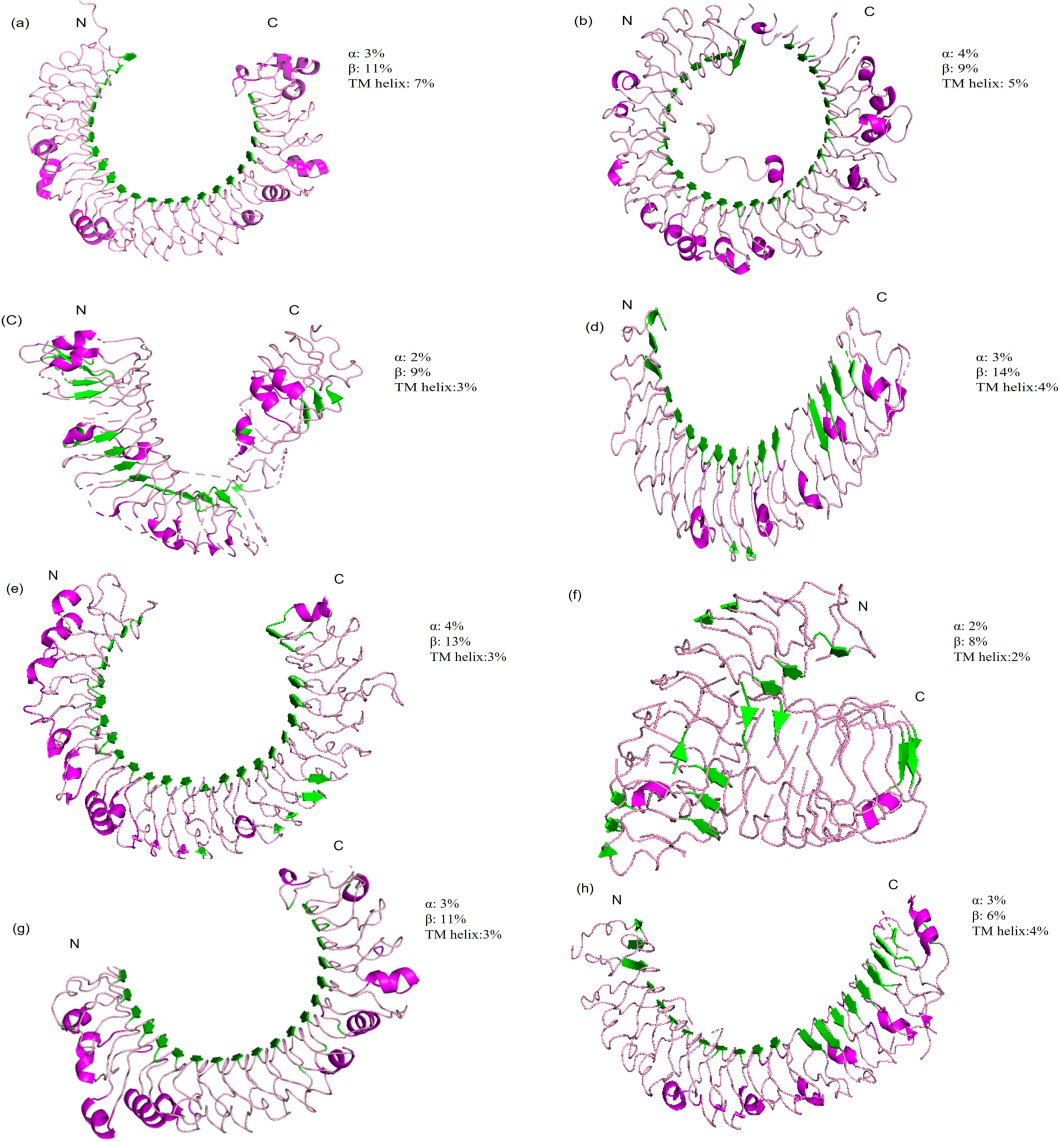
Three-dimensional structural models of leucine-rich repeat (LRR) regions in distinct (TLRs) in several fish species. In these models, α-helices are depicted in red tint, β-sheets in green, and turns in a pink. The N-terminal and C-terminal ends of the LRR domains are designated as LRR-NT and LRR-CT, respectively. **(A)** LrTLR1; **(B)** LrTLR2; **(C)** CcTLR9; **(D)** ToTLR23; **(E)** ToTLR27; **(F)** Tr LR22; **(G)** TrTLR26; **(H)** IpTLR27. Lr, Labeo rohita; Cc, Cyprinus carpio; To, Traachinotus ovatus; Tr, Takifugu rubripes; Ip, Ictalurus punctatus.

Other structural component of TLRs include α-helices and β sheets contributing vital role in conferring the ligands recognition and structural stability (8). Due to their significant role in fish Toll-like receptors, we have predicted 3D models (**Figure 2**) of different regions of LRR in TLRs by using a protein homology recognition engine (PHYRe, V2.0) (PHYRE2 Protein Fold Recognition Server (ic.ac.uk)) (25). For precise modeling of LRRs, emphasis was given to attaining maximum query coverage and sequence identity with confidence. Generally, 25-30% of query template sequence identity was considered reliable. All Leucine-rich repeat regions models have an acceptable coverage and confidence interval. The TLRs represented in **Figures 1**, **2** were carefully selected based on their importance in fish immunology research and their relevance to key aquatic species such as *Labeo rohita*, *Cyprinus carpio*, *Trachinotus ovatus*, *Takifugu rubripes*, and *Ictalurus punctatus*. These TLRs are integral to detecting and responding to pathogenic threats across these fish species, significantly enhancing our understanding of immune responses in aquatic organisms.

### 2.2 Classification of toll-like receptors in aquatic animals

To date, a total of twenty-one TLRs have been reported in aquatic animals, dividing into six families; (1) TLR1, (2) TLR3, (3) TLR4, (4) TLR5, (5) TLR7, and (6) TLR11 (26, 27). The family comprises of TLR1 subdivided in to subfamilies comprising on TLR1, TLR2, TLR6, TLR10, TLR14, TLR15, TLR16, TLR18, TLR25, TLR27, and TLR28. These subfamilies are responsible for identifying lipopeptides (28). For certain lipopeptides, members of the TLR1 family act as heterodimeric receptors. With other TLR1 family members, TLR2 always forms a dimer. The TLR2-TLR1 multimer recognizes lipopeptides containing a triacylated N-terminal cysteine. TLR2-TLR6 dimers are capable of identifying diacylated lipopeptides (29). TLR25 is structurally similar to TLR1 but it lacks the N-terminal cap and LRR (30, 31). Because of this, it has been suggested that TLR25 functions as a partner for forming heterodimers with TLR2 and TLR1, therefore encompassing the identification of a wider range of pathogens associated with molecular patterns as its ligands (32).

The family of TLR3, and TLR5 comprising on single member, while TLR4 and TLR5 have different variants. TLR4 is made up of TLR4a to 4d, whereas TLR5 is made up of TLR5a and 5b (33, 34).

The family of TLR7 comprises subfamilies of TLR7, 8, and 9. The primary function of these TLRs is to aid in the host’s defense against viruses. The Atlantic salmon (*Salmo salar*) contain TLR7, TLR8a2, and TLR8b2 (35), Atlantic cod (*Gadus morhua*) contain TLR7a and TLR9a (36), and common carp contain TLR7-1, TLR8-1, TLR8-2, and TLR8-3 (37). Unlike other vertebrates, several birds lack TLR8 and TLR9 (38), implying that the development of TLRs may have been significantly influenced by gene deletion.

The TLR11 family’s core categorization, which includes several frog and fish TLRs, is rather old. The subclass of TLRs11 comprising TLR11-13, TLR19-23 and TLR26 (39). Subfamilies of TLR11 have similarities to the TLR1 family (40, 41). Based on phylogenetic analysis, TLR20 has several subclades, including the catfish (*Siluriformes)* TLR20-1, TLR20-2 and TLR26. Phylogenetic analysis revealed that TLR26 could represent an early duplicated gene of TLR20 (36). However, precise TLR26 ligands in fish are yet unknown.

### 2.3 Evolutionary linages

**Figure 3:**
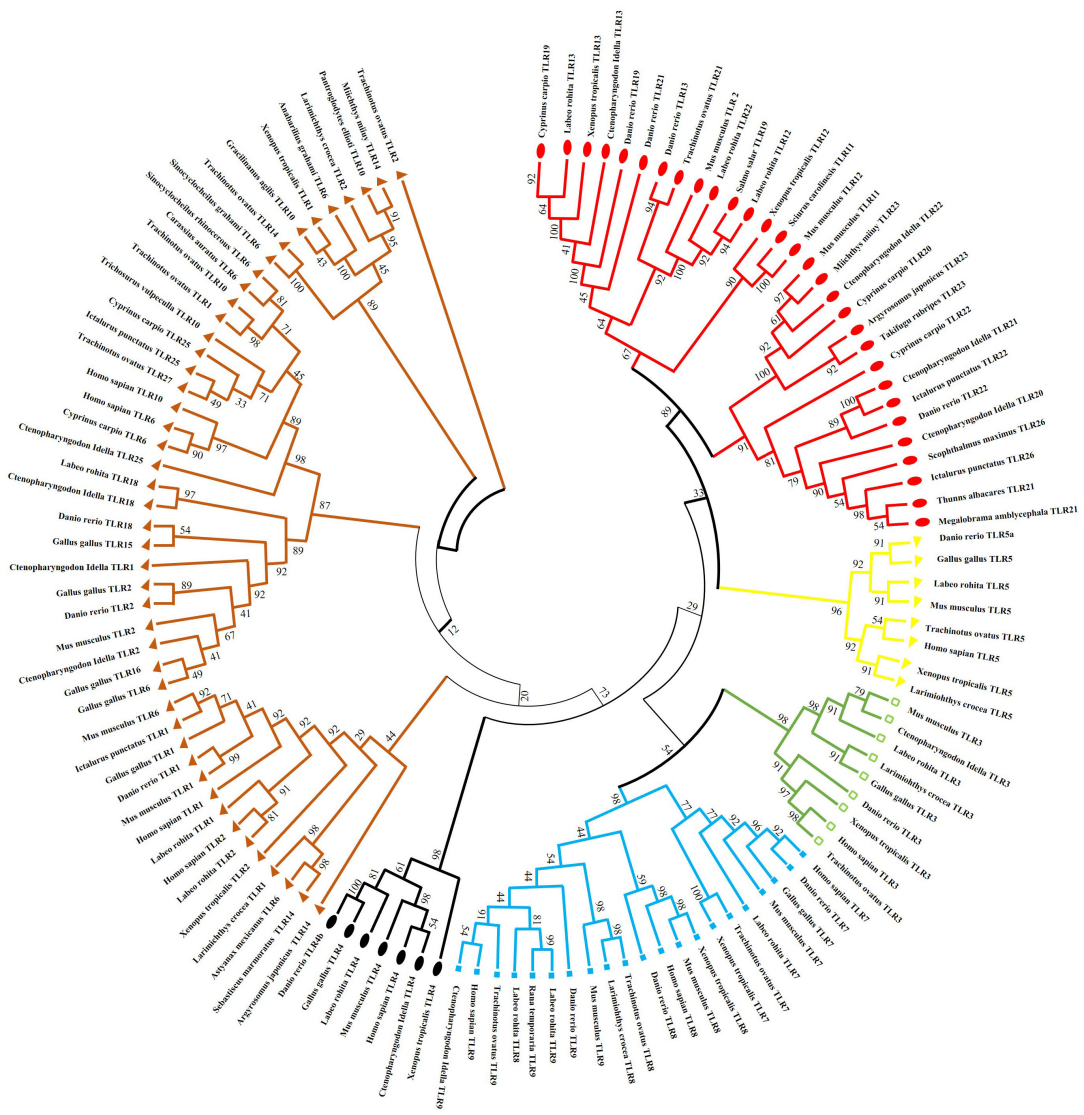
Phylogenetic relation of (TLRs) across different fish species, birds, amphibians and mammals. The complete amino acid sequences of various TLRs were obtained from GenBank. The consensus tree was constructed using the neighbor-joining method using MEGA7. The tree’s branches were confirmed through a bootstrap analysis based on 1000 replications, indicated by percentages at the branch nodes. Additionally, distinct TLR families are differentiated using various color codes.

Using the National Center for Biotechnology Information, a phylogenetic tree was constructed to explore evolutionary lineages using amino acid sequences of TLRs of fish, amphibians, mammals, and birds. GenBank sequence accession numbers of sampled species are presented in **Supplementary Table S1**. Tree validated that TLRs comprise six clades or families, and the results revealed some intriguing associations (**Figure 3**).

The subfamilies of TLR3 are present in the same clades and are more closely associated with TLR5 than TLR4. Subfamilies of TLR5 exist in the same clade and are more identical to the TLR11 family than the TLR7. Fish TLR1 is less closely linked to mammals and birds TLR1, TLR6, and TLR10 than it is to fish TLR1. After teleost TLR1 diverged from its corresponding progenitor gene, mammalian and avian TLR1 subtypes likely formed.

TLR18, which can be correlated with TLR14 in other species, is the homolog of TLR1 in humans found in zebrafish and channel catfish. The Nile tilapia (*Oreochromis niloticus*), fathead minnow (*Pimephales promelas*), medaka (*Oryzias latipes*), channel catfish (*Ictalurus punctatus*), and ayu (*Plecoglossus altivelis*) have all been shown to express toll-like receptor 25. TLR25 was first mistakenly identified as TLR1-like (28). However, our phylogenetic study showed that TLR25 is closely related and characterizes a recent new associate of the TLR1 family.

### 2.4 Nomenclature of TLRs

The naming complexity of TLRs in aquatic animals is influenced by the number of cysteine clusters in their extracellular Leucine-Rich Repeat (LRR) motif. They are classified as vertebrate type (V-Type) and protostome type (P-Type). V-Type TLRs, also known as Single Cysteine Cluster TLRs, contain a single cysteine cluster or CF motif in their carboxy-terminal domain (LRRCT) (21). In contrast, P-Type TLRs have multiple cysteine clusters or CF motifs, which can be located either on the LRRCT or the N-terminal domain (LRRNT). Few TLRs are designated as TLR4a, TLR5M, and TLR5S, reflecting various factors including gene duplication, alternative splicing, and evolutionary divergence. For instance, “TLR4a” indicates a specific isoform of the TLR4 gene, with “4” representing the TLR gene family and “a” distinguishing this variant. Similarly, “TLR5M” denotes the membrane-bound form of TLR5, while “TLR5S” refers to its soluble form. This naming convention is essential for differentiating between the various TLR forms and their functions. This classification helps in understanding the structural diversity and functional roles of TLRs across different aquatic species (42). Furthermore, Toll-like receptors are also named based on the organism from which they originate. For instance, LrTLR originates from *Labeo rohita*, and ToTLR originates from *Trachinotus ovatus.*

## 3 Signal transduction path

**Figure 4:**
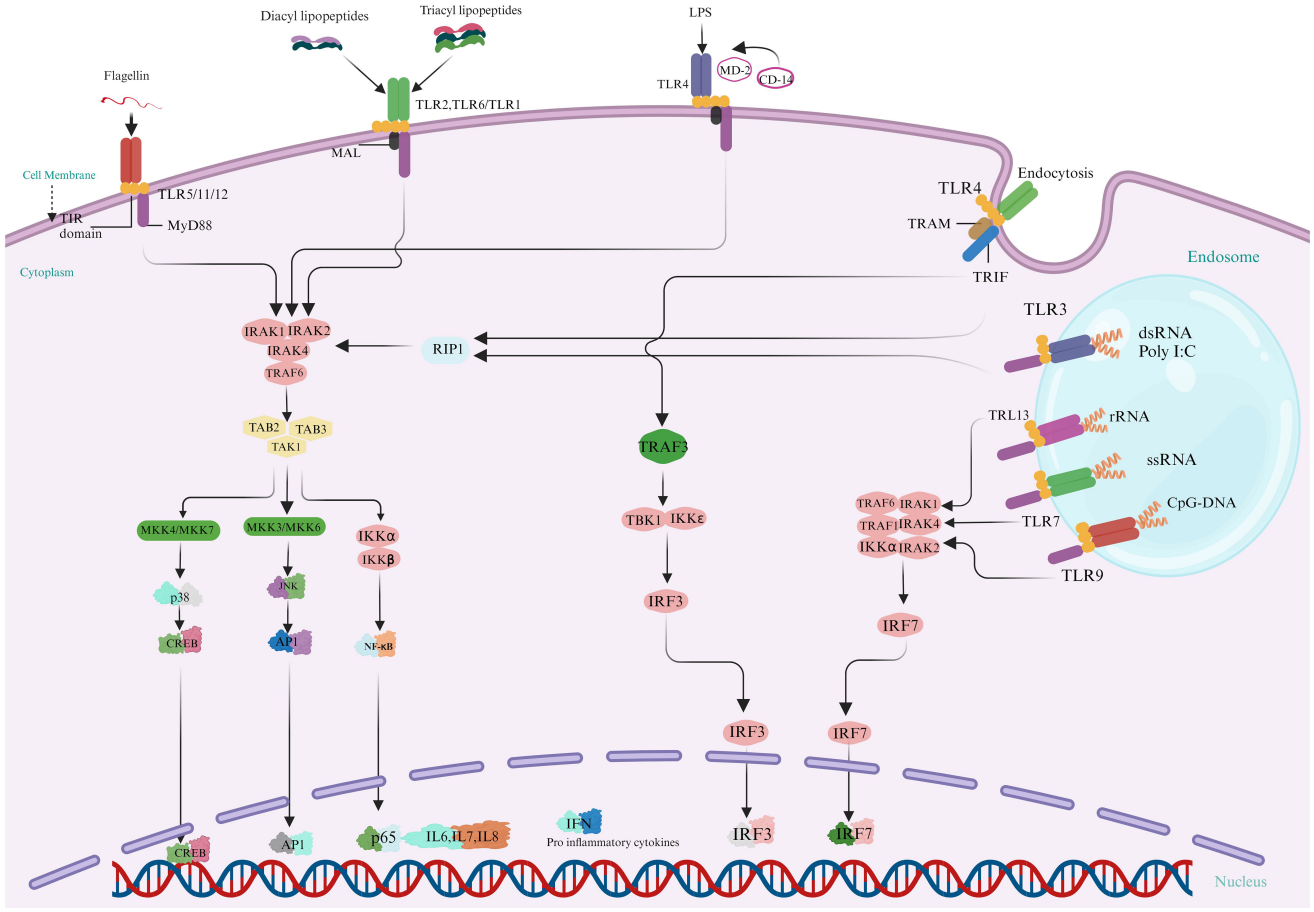
Activation of Multiple Toll-Like Receptor (TLR) Signaling Pathways leads to the synthesis of Innate Immune molecules: In the MyD88-dependent pathway, Myeloid Differentiation Primary Response 88 (MyD88) is a crucial adaptor in TLR signaling pathways in fish species. It activates downstream pathways such as NF-κB and MAPK, facilitating the production of proinflammatory cytokines by interacting with kinases and ubiquitin ligases. MyD88 is essential for TLR7 and TLR9 signaling, while TIRAP, another adaptor protein, aids in MyD88’s interaction with TLR2 and TLR4. MyD88-independent signaling, is important for TLR3 and partially TLR4, uses the TRIF/TICAM-1 pathway to produce IFN-β and activate IRF3 and NF-κB-1. This pathway supports diverse immune responses, with TRAM/TICAM2 linking TLR4 to TRIF in fishes. (Illustrations created with BioRender.com).

Toll-like receptors (TLRs), widely dispersed throughout aquatic animals, are critical for modulating immune cell proliferation and survival during infection (43). These proteins function as the primary defensive mechanism against various pathogenic attacks. Upon detection and binding to ligands, TLR signaling pathways activate, producing innate immunity molecules and recruiting adaptor molecules such as MyD88, TRIF, and MAL into the cytoplasm. Two distinct routes make the TLR signaling: (a) a system dependent on MyD88 and producing inflammatory cytokines (44) and (b) a MyD88-independent (TRIF-dependent) pathway is linked to the stimulation of IFN-β and the differentiation of dendritic cells. Generally, PRR-triggered signaling is routed via NF-kβ or related pathways, resulting in the host reaction through effector molecules. These effectors molecules comprise on AMPs, phagocytosis of hemocyte, encapsulation, and apoptosis. **Figure 4** displays the several TLRs’ signaling pathways and their effector molecules, interconnections, and downstream adaptive molecules.

### 3.1 Signaling via MyD88-dependent pathway

Myeloid differentiation primary response 88 is first identified and important signaling transducer commonly used by TLRs in aquatic animals (45). It is a vital adaptor protein that activates downstream signaling pathways used by all TLRs, with some exceptions. MyD88 pathway rapidly triggers transcription factors such as NF-κB, activator protein 1, and Elk-1, resulting in production of proinflammatory molecules like TNF-α, IL-6, and cyclooxygenase-2. This is how it functions in aquatic animals. MyD88 is drawn to TLRs upon identification of PAMPs. Stimulated MyD88 engage with the threonine/serine kinase interleukin-1 related kinase 4 protein. Following MyD88, IRAK4 is activated and is vital in triggering NF-Kβ and mitogen-activated protein kinase (MAPK). IRAK1 or IRAK2, another member of the IRAK family, is then activated by this complex. Subsequently, IRAK1 dissociates from MyD88 and interacts with TRAF6, a ubiquitin E3 ligase associated with the tumor necrosis factor receptor. An enzyme system that conjugates E2 ubiquitin made up of the E2 variant 1A (Uev1A) and E2 variant 13 (Ubc13) helps TRAF6 auto-ubiquitination by catalyzing the synthesis of K63 (lysine 63) associated multiple ubiquitin chains on TRAF6. TRAF6 interacts with several molecules, including TGF-β activated kinase 1 (TAK1), TAK1 adhesion molecules, TGF-β activated kinase TAB1, as well as TAB2 (46). TAB2 is an adaptor to link TAK1 to TRAF6, facilitating TAB1 activation (48). The complex of IKβ kinase, which consists of IKKα, IKKβ, and IKKγ/NF-Kβ, is activated by TAK1. Subsequently, IKKβ phosphorylates IKβ proteins connected to subunits of NF-Kβ, blocking nuclear trafficking. This leads to removing Kβ and permits NF-Kβ intranuclear shuttling. TNF-α, IL-8, and IL-12 are amongst the proinflammatory cytokines whose gene transcription is influenced by NF- Kβ constituents p50 and p65 (47, 48). By phosphorylating MAPKs, the MyD88-dependent cascade activates not only NF- Kβ but also the activator protein 1 (AP-1) complex, which aims the promotion of cytokine-associated genes. MyD88 is a necessary component of the signaling pathways of TLR7 and TLR9, which trigger the synthesis of IFN-I. TIRAP, also called adapter-like protein or MAL of MyD88, is an adaptor protein comprising a Toll/Interleukin-1 receptor (TIR) domain. It is essential for the interaction among TLRs and MyD88 in the signaling pathways of TLR2 and TLR4 (49, 50).

### 3.2 Signaling via MyD88-independent pathway

MyD88-independent signaling represents an alternative pathway that do not depend on the MyD88 adaptor protein to transduce signals (51). This pathway is especially crucial for providing a broader range of immune responses and exclusively utilized by TLR3 partially by TLR4. TRIF associated molecules (TICAM-1) plays an important role in this pathway, ensuring that the immune system can respond effectively to a wide variety of pathogens. In reaction to certain infections, TLR3 uses the TICAM-1/TRIF adaptor protein to initiate the production of IFN-β in a MyD88-independent manner (52). TICAM activates transduction factors IRF3 and NF-Kβ-1/TRIF (Adapter molecule 2 with TIR content), another downstream adapter via TLR4 relays signals, resulting in stimulation of a group of genes that encoding inflammatory cytokines and type I interferon (53). It has been shown that the splice variation of TRAM known as TAG (TRAM adaptor with GOLD domain) inhibits TLR4 signaling, which is independent of MyD88 (54). TLRs 3 and TLRs 4 activates IRF3 and IRF7, triggering antiviral responses. TRAM/TICAM2 connects TLR4 to TRIF in animals; however, it is absent in some fishes. Through TRAF3, TRIF activates TBK1, which phosphorylates IRF3 and activates the generation of type I IFN in conjunction with IKK. In vertebrates, TRIF’s NF-Kβ activation relies on an unknown association with RIP1, evading TRAF6; however, in mammals, TRIF interacts with both RIP1 and TRAF6 to accomplish (55–57). **Table 1** provides an updated summary of these processes as well as additional fish TLR characteristics.

**Table 1:** Summary of TLRs, their PAMP interface, downstream regulated molecules reported by experimental or in silico approaches, and references.

TLR	Member	Protein/PAMP	Signal/adaptor	Reference
TLR1	TLR1	Bacterial Flagella, triacyl-lipoprotein	TIRAP, SCIMP	(68)
	TLR2	Bacterial Flagella, triacyl-lipoprotein	MyD88, TIRAP, SCIMP	(68)
	TLR6	Lipopeptides conjugate	MyD88, TIRAP, SCIMP	(68)
	TLR10	Lipopeptide	MyD88	(145)
	TLR14	LTA, LPS, and zymosan	MyD88	(145)
	TLR18	dsRNA and LPS	–	(146)
	TLR25	dsRNA	–	(147)
	TLR28	dsRNA and LPS	SARM, SCIMP, TRIF, TICAM1	(148)
TLR3	TLR3	LTA, LPS, and zymosan	SCIMP, TICAM1, TICAM2	(68)
TLR4	TLR4	LPS	MyD88, TICAM1, TRIF	(50, 148)
TLR5	TLR5	Bacterial Flagella	MyD88	(68, 149)
TLR7	TLR7	ssRNA	MyD88, BCAP	(150)
	TLR8	ssRNA	MyD88	(140)
	TLR9	ssRNA	MyD88, BCAP	(151)
TLR11	TLR11	ssRNA	MyD88	(148)
	TLR13	Poly (I:C), 23S rRNA	MyD88, TIRAP, SCIMP	(152)
	TLR19	dsRNA, Poly (I: C)	–	(68)
	TLR21	CpG DNA	MyD88, TIRAP, SCIMP	(68)
	TLR26	LPS	–	(140)

ss, Single stranded; ds, Double stranded.

## 4 TLRs in innate immune system of aquatic animals

Developing enduring immunity in the organism and successfully removing invasive pathogens depends on robust innate immune responses (58). The innate immune system’s three constituents are the physical barrier, non-specific humoral response, and cellular components. Fish and higher-order vertebrates are the main species where these components’ role is still present (59, 60).

### 4.1 Physical barrier

Aquatic animals constantly interact with various pathogenic and non-pathogenic species in complex aquatic habitats, which sets them apart from terrestrial vertebrates. The skin of *teleosts* provides the preliminary defense against these infectious agents, the biggest immunologically active mucosal organ (61, 62). The most highly expressed TLRs in the skin of the infected fish are TLR1, 2, 5, and 5S, indicating that these TLRs could play a complex role in identifying Pathogens Associated Molecular Patterns of different aquatic animals diseases (63).

### 4.2 Non-specific humoral response

It is part of the innate immune system, involving humoral elements such as a variety of blood proteins and enzymes. Although these elements are not specific to any one pathogen, they play a vital role in combating infections. Macromolecules are discharged into extracellular fluids during invasions of various pathogens, which mediates the non-specific humoral response. The most studied non-specific macromolecules include liposomes, acute phase proteins (APP), antimicrobial peptides, and the complement system. When zebrafish contract *Aeromonas salmonicida*, they produce APPs through mechanisms driven by Toll-like receptors (TLRs). These receptors detect pathogen-associated molecular patterns during the infection. TLRs recognize PAMPs and trigger signaling pathways that lead to the production of cytokines such as IL-1, IL-6, and TNF-α. These proteins are crucial in enhancing phagocytosis, regulating immune responses, and promoting inflammation, thus playing vital roles in processes including inflammation and phagocytosis (64, 65). Lipopolysaccharide (LPS) functions as the classic ligand for TLR4 and is also widely recognized as an activator of the complement system. Similarly, zymosan, found in the cell walls of yeast, robustly activates the complement system and acts as a ligand for TLR2/6 in numerous fishes.

### 4.3 Cellular components

Fish neutrophils are the main fraction of granulocytes required for the innate immune response against various diseases. These leukocytes contain substantial antibacterial characteristics despite their short lifespan and intrinsic apoptosis (66). They release granules containing cytotoxic and antibacterial enzymes, among other extra and intracellular mechanisms (67). Except for TLR3, numerous Toll-like receptors (TLRs) are expressed by fish neutrophils. Neutrophils have been found to live longer when exposed to TLR agonists; *in vitro* and *in vivo*. Lipopolysaccharide priming significantly delays apoptosis. The host’s defenses against bacterial sepsis are greatly strengthened by delaying apoptosis (68). TLR2 agonists, on the other hand, have shown less dramatic effects on the suppression of neutrophil death, especially in populations of pure neutrophils (67).

Like their mammalian counterparts, rainbow trout dendritic cells (DCs) may phagocytose, react to Toll-like receptor ligands, express Dendritic cell marker genes (CD123, CD209) (69, 70), present molecules such as Major Histocompatibility Complex II, and migrate. Due to their ability to recognize double-stranded RNA (dsRNA), TLR3 and TLR22, which are present on trout skin CD8+ DCs, indicate that these cells are capable of identifying viral infections and then using cross-presentation to activate CD8+ T cells (13). Peripheral DCs are immature cells with a high endocytic capability for effective antigen absorption. They can develop when started by different microbiological components and express numerous TLRs, including TLR1, 2, 4, and 5. Inflammatory cytokines like IL-12 and co-stimulatory molecules like CD80/CD86 are expressed by dendritic cells in response to TLR-mediated detection of microbial components. DCs that have activated TLR4 or TLR9 produce IL-12, which affects T helper (Th) cell development into Th1 cells (71, 72). TLR signaling in dendritic cells plays a crucial role in regulating the Th1/Th2 balance in mice, as evidenced by the Th1 and Th2-type responses elicited by LPS from *P. gingivalis* and *E. coli*, respectively (73, 74).

## 5 TLRs in adoptive immune system of aquatic animals

Adaptive immunity is the body’s second defense against invasive infectious agents (75). Pathogen proliferation can be inhibited or eliminated by involving specialized systemic cells and processes. The innate immune response triggers two main constituents of the adaptive immune system. These are lymphocytes, essential for the cell-mediated immunological response (76).

B and T lymphocytes are vital components of adaptive immunity. These specialized lymphocytes ‘ essential functions include the recognition of pathogens and the instigation of adaptive immune responses (77, 78). Primary humoral immune response cells that produce antigen-specific antibodies and memory cells that last throughout time are produced by B-cells. On the other hand, T-cells are primarily found in the mucosal tissues of fish, including the gut and gills. Immunoglobulins (Igs), such as IgM, IgD, and IgT (also called IgZ in *cyprinid* fish) (79), are synthesized in fish by the action of B cells. The first teleost Ig to be defined is IgM, regarded as a universal vertebrate Ig (48, 80).

Conversely, IgT is a special teleost Ig that mainly functions in mucosal immunity. Besides their Ig receptors, fish B cells may also use other pattern recognition receptors (PRRs), such as Toll-like receptors (TLRs), to directly identify and react to infections (81). For example, IgM+ B cells in salmon continuously express nucleic acid-sensing TLRs. These B cells generate IFN-I in response to ds/ssRNA and CpG DNA stimulation (82). In addition, R848, a ligand for TLR7/TLR8, dramatically boosts the production of IFNα1 and IFNβ (83). While TLR3 is low in trout, TLR8α2, TLR9, and TLR22 are more prevalent in Immunoglobulin M+ B cell populations found in the blood, kidney, and spleen. TLR9 expression is upregulated after stimulation in rainbow trout and Atlantic salmon studies (84).

## 6 Expression pattern and pathogen associated immune response

TLR expression is controlled and changes according to the pathogen type and immune cells involved. Different TLRs can identify particular PAMPs, including double-stranded RNA (dsRNA), lipopeptides, and lipopolysaccharides (LPS) (84, 85). For instance, TLR4 can remember LPS from Gram-negative bacteria, but TLR3 is known to recognize dsRNA that is generally generated during viral infections. Several pathogenic invasions cause an increase in TLR gene expression in a variety of aquatic species. The expression of the TLR gene is stimulated in goldfish macrophages when exposed to *Mycobacterium chelonae*. Following mycobacterium infection, the zebrafish exhibits upregulation of the TLR1, TLR5, TLR18, TLR20, and TLR22 genes. Nonetheless, zMyD88, zTRIF, and zSARM expression levels remain unchanged (86).

Important Toll-like receptors (TLRs), including TLR2, TLR3, TLR4, TLR9, TLR20a, and TLR22, are overexpressed in channel catfish due to infections such as *Edwardsiella ictaluri* (87). These toll-like receptors are essential constituents of the immune system that help the catfish survive and fight against enteric septicemia (88). TLR20 and TLR21 are also expressed more in the spleen, kidney, and gut in response to infections such as *Aeromonas hydrophila*. *Ictalurus punctatus* tissues infected with *Ichthyophthirius multifiliis* also contain TLR21 (89). To protect against infections, these TLRs trigger signaling cascades upon identifying pathogen-specific epitopes. Essential immune genes such IFN, MX, Gig, and IL-1β are expressed in grass carp due to signaling pathways that are started by recognizing the grass carp reovirus (GCRV) by Toll-like receptor 3 (TLR3) (90). TLR3 mRNA expression also increased in response to GCRV infection in guppy fish (*Poecilia reticulata*), suggesting that TLR3 plays a role in the antiviral response (91). TLR2 can identify peptidoglycan and lipoteichoic acid, two components of the *Staphylococcus aureus*, in Zebrafish (*Danio* rerio) (92). However, it is less sensitive to certain synthetic lipopeptides. The immunological responses that the carp has against particular infections dependent on these TLR interactions.

TLR2 expression is upregulated in carp species such as mrigal and rohu in response to infections caused by differentially stained bacteria, peptidoglycan (PGN), and lipoteichoic acid (LTA) stimulation. The grass carp’s TLR4 gene expression increases in the liver and muscle tissues infected with GCRV, suggesting it protects against viral infections (19). TLR5 gene expression in mrigal rises in response to *Aeromonas hydrophila* infection in particular organs (93). When the flagellin of *Vibrio anguillarum* is bound by the gilthead seabream fish, TLR5 is involved in both the membrane form (TLR5M) and the soluble form (TLR5s), which in turn causes an inflammatory response (94). An overview of the pathogens that cause illnesses in fish and the particular organs where certain TLRs become activated after infections is presented in **Table 2**.

**Table 2:** Response of toll-like receptors to different pathogens.

Pathogen	Exogenous Ligand	Host	TLR Molecule	Organ	Reference
Virus	*Salmon anemia virus*	*Salmo salar*	TLR3	Brain & kidney	(37)
	*Grass carp reovirus*	*Gobio cypris rarus*	TLR5	Gills, Muscle & spleen	(153)
	*Grass carp reovirus*	*Ctenopharyngodon* *Idella*	TLR3, & 22	Gills & spleen	(154)
	*Koi herpes virus*	*Cyprinus rubrofuscus*	TLR3	Kidney & liver	(155)
	*Viral hemorrhagic septicaemia virus*	*Paralichthys olivaceus*	TLR14	Kidney	(156)
Bacteria	*Streptococcus uberis*	*Cirrhinus mrigala*	TLR2	liver & Intestine	(157)
	*Vibrio anguillarum*	*Sparus aurata*	TLR5S	Thymus	(98, 100)
	*Aeromonas hydrophila*	*Nibea albiflora*	TLR2	Kidney & Liver	(158)
	*Vibrio aliginolyticus*	*Cirrhinus mrigala*	TLR13	Muscle & spleen	(112)
	*Aeromonas hydrophila*	*Clarias magur*	TLR22	Spleen	(135)
Parasite	*Argulus siamensis*	*Nibea albiflora*	TLR2	Kidney	(159)
	*Cryptocaryon irritans*	*Catla catla*	TLR 22	Spleen	(160)
	*Trypanosomes*	*Labeo rohita*	TLR13	Kidney & Liver	(126)
Fungi	*Microsporidians*	*Sparus aurata*	TLR2, 5b	Thymus	(161)
	*Saprolegnia parasitica*	*Cirrhinus mrigala*	TLR5	Gills & Muscle	(27, 162)

## 7 Variability in TLR expression across aquatic animals

### 7.1 Shellfish

Shellfish species like *Macrobrachium rosenbergii*, *Penaeus monodon*, and *Liptopaenus vannemi* are economically important and commonly studied in aquaculture due to their high market demand, rapid growth rates, and global commercial value. These species are particularly favored in the aquaculture industry for their adaptability to various farming environments, including freshwater and marine systems. These species not only strengthen aquaculture production but also serve as model organisms in environmental and biological research. However, they face significant challenges due to susceptibility to various diseases, which can severely impact production. Studies on TLRs are crucial for understanding immune responses and developing effective disease prevention and management strategies, contributing to healthier and more sustainable aquaculture practices.

#### 7.1.1 Giant freshwater prawn (*Macrobrachium rosenbergii*)

This prawn is native to tropical and subtropical regions of South and Southeast Asia. Two distinct toll-like receptors have been discovered in freshwater prawns and categorized as type-1 and 2 crustacean TLRs (95, 96). These genes are expressed in several organs of *M rosenbergii*, but the expression is more significant in gills. MrToll1 and MrToll2 regulate the expression of MyD88, prophenoloxidase (proPO), and AMPs (97). The expression of anti-lipopolysaccharide factor (ALF) was upregulated in prawns with silencing of MrToll1 and MrToll2 but downregulated in those with gene silencing of MrToll3. It indicates an essential role of TLR in the immune functioning of prawns (98).

#### 7.1.2 Shrimp (*Penaeus monodon*)

Black tiger shrimps (*Penaeus monodon*) are marine crustaceans widely distributed in Asia. The PmToll is a type I protein discovered in 2007 in *P monodon* (21, 99). It initiates an immune response leading to the activation of the NF-kβ pathway following infection with poly I: C or white spot syndrome virus. Some studies indicate that a variant of this receptor, PmToll9, could initiate an immune cascade that stimulates the NF-kβ. In shrimps infected with *Vibrio Hervey*, there is increased expression of PmToll in the gills, midgut, and stomach. Moreover, it has been found that microbial challenges lead to a noticeable increase in PmTRAF6 and PmMyD88 in lymphoid organs and hepatopancreas.

#### 7.1.3 Pacific white shrimp (*Liptopaenus vannemi*)

The Pacific white shrimps’ genome is reported to contain 11 TLR genes. Toll 4 is essential for immunity against WSSV infection. TLR expression genes are principally reported in the muscle, hemocytes, hepatopancreas, stomach, and intestine of *L. vannemi* (100). Specific defense against Gram-positive bacteria is enhanced by alteration in single nucleotide polymorphism in the LvToll1 gene, which becomes more noticeable during infections. However, the impact of these modifications on Gram-negative bacteria and the White Spot Syndrome Virus is reduced. This implies that these genetic variants impact on immune response (101). The intricacy of immune system interactions is shown by the efficiency changes depending on the kind of infection when subjected to infections from *V. parahaemolyticus*, *S. iniae*, WSSV, fragments from the *L. vannemi*. Toll3-LRR-CT produced 9, 7, and 7 additional bands.

### 7.2 Molluscs

Molluscs such as Pacific oyster (*Crassostrea gigas*) and mud crab (*Scylla paramamosain*) play vital roles in both aquaculture and research, owing to their significant ecological functions and economic value. *Crassostrea gigas* is one of the most extensively farmed oyster species worldwide, contributing to ecosystem services such as water filtration, habitat creation, and nutrient cycling in coastal ecosystems, while also supporting global seafood markets (102). Similarly, *Scylla paramamosain* is an important species in crab aquaculture, valued for its high meat quality and growing market demand in Asia.

#### 7.2.1 Pacific oyster (*Crassostrea gigas*)

This species possesses 83 TLR genes, including four P-type, five V-type, and seventy-four mixed types. Overexpressing V-type TLRs in human kidney embryonic cells stimulates NF-Kβ signaling (103). In reaction to an infection with *V. anguillarum*, P-type cgToll-1 was triggered, while *C. gigas* hemocytes expressed five V-type TLRs after being exposed to heat-inactivated viral particle. Compared to other mollusc LRRs, CgToll-3’s LRRs are more preserved since they are premeditated to identify *Vibrio* sp. Early in the embryonic stage, *C. gigas* has increased TLR expression, which is essential for defense against infection (28).

#### 7.2.2 Mud crab (Scylla paramamosain)

This crab species has been shown to possess SpToll1 and SpToll2, two distinct toll receptors. SpToll2 is closely related to *Eriocheir sinensi* Toll2 and *Portunus trituberculatus* Toll4, forming groups with Tol l1, Toll3, and Toll4 of *Drosophila melanogaster* (104). It’s a form between a V-type toll and a P-type. The expression of the SpToll and SpMyD88 genes upsurges in response to *V. harveyi* infection. However, only one gene whose expression is increased in response to *V. parahemolyticus* and *S. aureus* infections is the SpMyD88 gene. Additionally, it has been shown that in crabs, SNPs in the SpToll gene are associated with amplified resistance to infection (105).

### 7.3 Major carp

Major carps, including Mrigal carp (*Cirrhinus cirrhosus*), Rohu (*Labeo rohita*), and Catla (*Labeo catla*), are widely cultivated freshwater fish species. These species are highly valued for their nutritional quality, rapid growth, and adaptability to diverse freshwater ecosystems, making them essential components of polyculture systems. Research on TLRs in major carp species is critical to improving disease resistance, immune response understanding, and overall health management in aquaculture. By exploring the genetic and molecular mechanisms of TLRs, researchers can enhance the resilience of carp to common infections and environmental stressors.

#### 7.3.1 Mrigal carp (*Cirrhinus cirrhosus*)

The mrigal carp also called white carp, is one of the most popular fish for commerce. Several vital organs for the immune system in this kind of fish showed increased IL-10 and TLR4 gene expression in response to *A. hydrophila* infection and LPS stimulation. Studies have shown that following bacterial challenges and PGN stimulation, the gut and kidney of mrigal exhibit a strong relationship between the upregulation of TLR2, activation of MyD88 and TRAF6, TNF-α, and IL-8 gene production (106).

#### 7.3.2 Rohu (*Labeo rohita)*

The species of this carp is a vast, silver-colored fish native to Southeast Asia. Various bacterial challenges and zymosan stimulations triggered TLR2-signaling pathways, which led to the production of IL-8 and then activated the downstream signaling molecules (107). The critical component of gram-negative bacteria’s cell walls, LPS, is recognized primarily by TLR4. *A. hydrophila* infection in rohu produced pro-inflammatory type I IFN and cytokines by activating TLR4 and starting signaling pathways that are dependent on TRIF and MyD88 (108). Additionally, it has been demonstrated that TLR3-activated signaling triggered the NF-Kβ signaling pathways, producing IL-1β gene expression and TNF-α.

#### 7.3.3 Catla (*Labeo catla*)

This species usually called the omnivorous carp of South Asia, is an economic fish. The TLR2, TLR5, and TLR6 genes are widely expressed in all physiologically vital organs of fish. Robust expression of the TLR2 and TLR6 genes in the kidney suggests that the kidney plays vital roles in innate immune and hematopoietic functions in the lack of bone marrow. *S. uberis* infection and PGN stimulation activate TLR2, whereas poly I: C activated TLR5 genes. Moreover, the activation of TLR4, MyD88, and TICAM1 by LPS demonstrated the function of both MyD88-dependent/independent signaling pathways (109).

### 7.4 Bony fishes

Species of Bony fishes such as are highly popular in the global aquarium trade, valued for their vibrant colors, diverse patterns, and adaptability to captive environments (110). These species are not only economically significant in the fish industry but also hold considerable value as model organisms in biomedical research. Additionally, their use in environmental toxicology helps assess the impact of pollutants on aquatic ecosystems, highlighting their dual importance in both commercial and scientific contexts.

#### 7.4.1 Ornamental fishes

##### 7.4.1.1 Koi carp (Cyprinus rubrofuscus)

This carp is commercially important farmed ornamental fish species, displaying patterns in gold, orange, silver, white, and black hues. Type I interferon, MX3, and TLR9 genes were expressed in juvenile koi carp due to an infection with bacteria. The highest homology was found between the amino acid sequences of ccMyD88a and MyD88b and those of zebrafish, channel fish, and mammals. The TRAF6 gene is primarily expressed in the gills and liver, and the Max dimerization protein 3 gene is substantially increased inside the kidney after koi *ranavirus* (KIRV) infection (111).

##### 7.4.1.2 Goldfish (*Carassius auratus*)

It is a commercially important, elongated, and stocky golden color fish. It is frequently used experimentally because of its accessibility. The 1st TLR has been found in macrophages of LPS-stimulated gold fish. Spleen and kidney tissues revealed a significant upregulation of TLR2, TLR3, and TLR7 due to bacterial challenge. There was modulation in the TLR3, 4, 9, and TLR22 pattern against infestation of *Argulus* sp. TLR4, 20, and TLR22a against infestation of *D intermedius* (112). It has also been discovered that TLR5 and 13 exhibits markedly elevated expression during an acute mycobacterial infection.

##### 7.4.1.3 Zebrafish (*Danio rerio*)

This is a small-sized freshwater fish and a popular model organism for studying the innate immune system. Most Pattern recognition receptors and their associated molecules found in vertebrates have also been found in zebrafish (92). TLRs are predominantly present in the skin, suggesting a significant role in defending against pathogens. Nearly all of the approximately twenty possible TLR variants exist in zebrafish. Among these, ten are human TLR orthologs. TLR20, similar to TLR11 and TLR12 in mice, has been detected in common carp and zebrafish. In contrast to other species, TLR4 does not react to lipopolysaccharide (LPS) in zebrafish (113). Additionally, TLR4 may have different functions in pathogen detection if TLR4a, TLR4b, and MyD88 are suppressed.

#### 7.4.2 Commercial fishes

##### 7.4.2.1 Pangas catfish (*Pangasius Pangasius*)

The Pangasiid catfishes consist of several related species that are widely recognized in the market as pangasius. They are primarily found in Vietnam and other parts of Southeast Asia. The tissues from various organs, including the brain, gill, liver, intestine, and kidney of *Pangasius pangasius*, were examined using semi-quantitative RT-PCR. Upon analysis of differential expression, TLR4 exhibited the most significant expression, whereas TLR2 showed the lowest (114).

##### 7.4.2.2 Striped catfish (*Pangasius hypophthalmus*)

The Iridescent catfish is indigenous to Southeast Asian rivers and is also found in the Mekong basin, where it is intensively raised for food. Four distinct PRR families, including C-type lectin receptors and TLRs, along with cytosolic proteins such as Retinoic acid-inducible gene (RIG) and NOD-like receptors, are crucial for sensing diverse stimuli in striped catfish. The expression of the soluble form of TLR5 was examined in *Pangasianodon hypophthalmus* in response to *Edwardsiella tarda* infection, which recognizes bacterial flagellin and activates innate immune responses (115). The study identified significant TLR5S expression in the spleen at 24 hours post-infection and in the kidney at 72 hours, indicating that the kidney provides extended protection during the immune response (116).

### 7.5 Anabas

Anabas species, commonly known as climbing gourami, can be found in both freshwater and brackish zones in India, Bangladesh, Sri Lanka, and China. They possess a specialized respiratory system in their head that enables them to absorb atmospheric oxygen, allowing them to survive out of water for prolonged durations. A total of 16 TLR genes were identified and characterized in *Anabas* species. The TLR genes of *Anabas* were classified into five subfamilies (TLR1, TLR3, TLR5, TLR7, and TLR11-subfamily) based on phylogenetic analysis, with their annotations confirmed through syntenic analysis. Through the comprehensive annotation of the integrated unigene dataset using KEGG pathways in Siamese fighting fish (*Betta splendens*), several key genes associated with innate immunity were identified. This includes 55 genes related to TLRs, highlighting that TLRs play a significant role in the immune system of fish species belonging to the Anabantiformes (117).

## 8 Influence of biotic and abiotic factors on TLR functionality in aquatic species

### 8.1 Biotic factors

Biotic factors, such as pathogens and parasites, interact with TLRs to shape immune response mechanisms in aquatic species (118). TLRs represent the most ancient class of receptors, possessing the broadest range of pathogen recognition. Numerous studies suggest that TLR2, TLR5M, TLR5S, TLR9, and TLR21 may specifically recognize PAMPs from bacteria. Additionally, other TLRs, such as TLR1, TLR4, TLR18, and TLR25, may also serve as bacterial sensors. TLR signaling pathways in fish display distinct characteristics that differ from those in mammals.

An experiment conducted by administering poly(I:C) and *Aeromonas hydrophila* injections revealed that TLR19 expression was significantly upregulated in the immune-related organs of *Cyprinus carpio L*. Immunofluorescence and luciferase analyses showed that TLR19 recruits TRIF as an adaptor and can activate the expression of interferon-1 and viperin. These findings suggest that TLR19 plays a crucial role in the innate immune response of aquatic species, particularly in antiviral and antimicrobial defense mechanisms (119).

### 8.2 Abiotic factors

Abiotic conditions like as temperature shifts, salinity alterations, pH changes, and levels of oxygen are typical in aquaculture environments. Temperature variations can significantly influence the expression and functionality of TLRs in fish, as it affects their metabolic rate, immune system efficiency, and susceptibility to pathogens (9, 120–124). For example, in *Atlantic salmon*, it has been observed that lower water temperatures can lead to reduced TLR expression, weakening their immune response to bacterial infections like *Aeromonas salmonicida*. Conversely, higher temperatures may enhance TLR activity but could also increase the fish’s vulnerability to stress and disease.

The study investigated the effects of nickel (Ni) and high temperature (Ni+T) on Pangasianodon hypophthalmus. Exposure to Ni and Ni+T significantly increased oxidative stress markers and altered gene expression related to immune response, metabolism, and stress in the fish. Ni bioaccumulation was highest in the kidney and liver, with DNA damage observed in gill tissue. Despite some depuration after 28 days, Ni levels in tissues remained concerning, indicating long-term residual effects (125).

The study examined the effects of 16-hour simulated transport stress on hybrid yellow catfish, followed by a 96-hour recovery, finding that initial stress increased alkaline phosphatase and antioxidant activity, which later decreased. Transcriptome analysis further identified 1,525 differentially expressed genes, underscoring the role of Toll- and NOD-like receptor pathways in the observed stress response. While some recovery occurred after 96 hours, a longer recovery period is suggested. The findings offer insights into stress-induced immunoregulation in aquaculture. Under severe abiotic conditions, TLR activation may result in the synthesis of heat shock proteins (HSPs) and other stress-related molecules that protect fish cells from damage. These defensive mechanisms underscore TLRs’ dual involvement in pathogen defense and stress management, making them critical for sustaining fish health in varied and often harsh aquaculture conditions.

## 9 Strengthen the spawn resilience through immune training

When the adaptive immune system is still developing in spawns, training the innate immune system could increase its ability to resist an array of aquatic diseases. This approach shows much potential for intense larval production. Aquaculture has long employed immune stimulants and TLR-ligand supplementation to stimulate fish’s non-specific innate immune responses (126). Considering the outlined process, immunological treatment can strengthen fish’s natural defenses, especially against viruses. This strategy opens the door for creative approaches to raising fish larvae.

Initially, fish larvae that have just hatched might be treated with substances that trigger trained innate immunity using bath treatment employing specific Pathogen-Associated Molecular Patterns. This can trigger the formation of interferons and interleukins by activating a variety of Pattern Recognition Receptors (127, 128).

Secondly, only a small dosage of PAMPs can be used to boost the innate immune systems of male and female larval fish. This strengthens their resistance to current illnesses and permits the transfer of this enhanced innate immunity to their progeny (F1 generation). The process by which tolerance from both parents is transferred to the young has been confirmed in the pipefish (*Syngnathus typhle*).

Thirdly, fish growth is significantly impacted by the early diet. Fish eat zooplankton, algae, or specially prepared meals once the yolk is depleted. During development, immune stimulants with various TLR ligands/PAMPs can be supplemented to the diet to trigger the PRRs-signaling pathways of fishes. This stimulation offers protection against a broadly distributed pathogen (129).

## 10 TLR based strategies for diseases management in aquaculture

To combat infectious diseases in aquaculture, it’s crucial to adopt strategies based on Toll-like Receptors. For example, in Atlantic salmon and rainbow trout, the activation of TLR7 and TLR8 stimulates responses in essential immune related organs like the brain, kidney, and spleen, all of which express a diverse range of TLRs (31, 130). They represent critical elements in the formulation of effective vaccines. Upon exposure to TLR ligands, these organs generate type I interferons and pro-inflammatory cytokines, crucial for initiating immune responses against pathogens. TLR ligands, including LPS as a TLR4 ligand and its less toxic derivative Imiquimod (MPL), along with poly I:C as a TLR3 ligand, are vital for stimulating the immune system of aquatic animals.

Pre-treatment with poly I:C in zebrafish has proven effective in delaying mortality onset caused by the viral hemorrhagic septicemia virus (VHSV), a significant pathogen in aquaculture (131, 132). Furthermore, the activation of TLR9 by bacterial infections in zebrafish, where TLR9 identifies CpG ODNs, showcases an additional immune activation layer aiding in bacterial threat management. This mechanism stimulates the NF-κB pathway, crucial for inflammatory and immune responses. Additionally, the identification of viral dsRNA by TLR22 in fugu, underscores the evolutionary adaptation and specialization of TLR in fish for detecting and responding to viral infections.

These insights underscore the vital role of TLRs in immune regulation against infections, highlighting their potential as targets for disease management strategies in aquaculture.

## 11 Aquatic animals vs. mammalian toll-like receptors: a comparative analysis

Mammalian Toll-Like Receptors have been extensively investigated, revealing that they are essential in strengthening the body’s defense against many illnesses. Numerous non-mammalian species, such as amphibians, reptiles, fish, and birds, have had TLRs or related proteins in their genomes (133). Although these TLRs frequently display traits analogous to those seen in mammals, they vary from their counterparts in specific ways. TLR2 in mammals’ forms homodimers or heterodimers with TLR1 or TLR6 to recognize pathogenic PAMPs. The absence of LRRNT in TLR1 is essential for this dimerization process (134). Therefore, it is possible that, in line with the mammalian framework, piscine TLR1 may likewise participate in synthesizing dimers with TLR2. Non-mammalian vertebrates have TLR2-6 heterodimers that detect MALP-2. However, the absence of TLR6 in the fish gene may account for their insensitivity to MALP-2 (21, 135).

LPS, a crucial element of Gram-negative bacterial membranes, is recognized by mammalian TLR4. Not every species of fish has TLR4, in contrast to humans. In contrast to mammals, fish and amphibians have a high tolerance to LPS and require significantly higher dosages to stimulate their leukocytes. Auxiliary molecules such as MD2, CD14, and LBP, essential for identifying LPS in mammals, are deficient in some essential features in fish (136). For example, several mammalian characteristics necessary for binding to LPS are absent from channel catfish TLR4. These variations suggest that fish and humans have different mechanisms for recognizing LPS.

Mammals have a receptor TLR5, which is essential for detecting flagellin, which is part of bacterial flagella. In fish, there are two versions of TLR5. While TLR5S is specific to fish and does not have certain domains, TLR5M is identical to TLR5 in mammals (137). Certain species, such as Japanese flounder, fugu, channel catfish, and rainbow trout, have both variations. Flagellin triggers a feedback process in which activation of TLR5M results in the synthesis of TLR5S and subsequent cellular reactions. The precise function of TLR5S is still unknown (138).

In mammals, TLR6 and TLR8 have been shown to be activated by immunomodulators imidazoquinoline derivatives with antiviral properties and are involved in detecting single-stranded RNA (139). However, in fish, there has not yet been direct evidence to confirm ligand specificity for these receptors. Additionally, the expression of chemokines in response to resiquimod (R848) was not inhibited by chloroquine, suggesting significant variation in the localization or stimulation of TLR7/8 between mammalians and aquatic animals (140). The surface features of outer or inner membranes do not activate TLR3, TLR8, and TLR9, in contrast to other TLRs, which are activated by microbial nucleic acids. Human TLR9 selectively identifies the CpG DNA, which is widely present in prokaryotes but seldom in eukaryotes. Therefore, how TLR9 binds to ligands in fish needs to be clarified (141, 142). used qRT-PCR to quantify the gene expression of TLR9 in cobia (*Rachycentron canadum*) following CpG-ODN stimulation. The findings revealed that, compared to the untreated groups, the CpG-ODN-treated groups exhibited increased levels of proinflammatory cytokines and TLR9 in their spleen and liver. The TLR9 gene was shown to be expressed in many organs and at all developmental stages. The ubiquitous expression of the TLR9 gene implies that it may have a protective function in the initial phases of development, mainly when infections are a significant hazard to fish larvae (126).

## 12 Conclusion and prospects

The need for healthy and safe food supplies increases as the global population expands. This necessity is met mainly by fish and other creatures of the water, particularly in areas with little access to alternate protein sources. Millions of people worldwide depend on the fishing and aquaculture sectors for their livelihood (143). However, fish susceptibility to diseases and infections is a significant obstacle to the long-term cultivation. This vulnerability is especially noticeable during the incubation period of eggs in incubators. *Saprolegniasis*, also called a cotton wool disease, poses a severe risk to the endurance of seeds, fry, and fingerlings in many aquatic species, including trout and carp. It is characterized by cotton-like growth on fish eggs. Furthermore, the longevity of fingerlings and adult fishes is threatened by parasite infections such as dermatosis, monogeneans, and trichomoniasis. *L. rohita, C. idella, C. catla, C. mrigala, C. carpio*, *H molitrix*, and *O mossambicus* are among the susceptible species.

**Figure 5:**
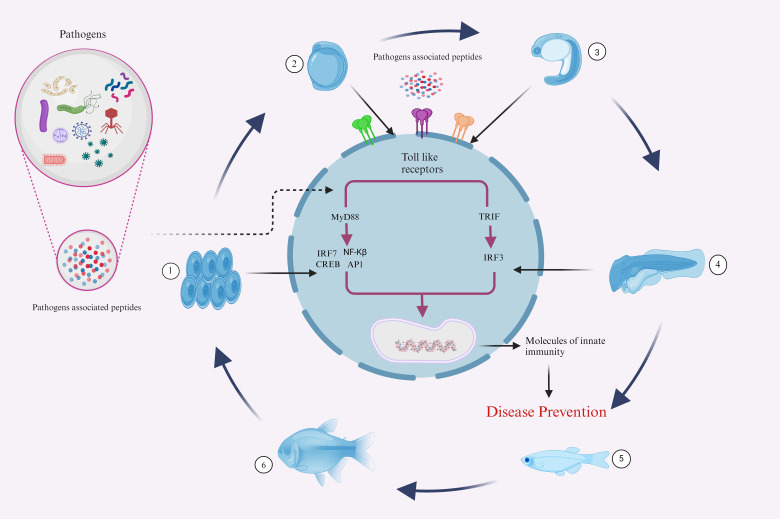
Illustrative representations of fish complex innate immune responses to several infectious agents. The developmental Phases 1-6, representing the Embryo, yolk sac larva, pre-larva, larva, juvenile, and adult stages, demonstrate the sophisticated defense mechanisms that aquatic animals employ against a variety of pathogens throughout their life cycle. (Illustrations created with BioRender.com).

Fish’s innate immune system is their initial line of defense against pathogen invasion. This process is mediated by numerous factors, including TLRs, which trigger IFN and cytokines production through MyD88-dependent or independent pathways, influencing the immune system and inflammatory process (**Figure 5**). The investigation of fish TLRs has advanced significantly in the past few years, primarily due to identifying and characterizing fish’s innate immune receptors. From an immunological perspective, these TLRs might be targeted by administering agonists or ligands that are specific to them, which can interact with and activate them, functioning in a vaccine-like manner (143, 144). In order to fully utilize TLR-based techniques, current and subsequent research must focus on specific crucial goals. First, it is critical to have an in-depth comprehension of the complex signaling pathways triggering the fish TLRs.

Secondly, investigation is needed to pinpoint the precise ligands that fish TLRs can bind to elicit immunological responses. Thirdly, there is a need to explore the distinct immunological profiles of various fish populations. Fourthly, using TLRs to generate vaccines is an intriguing strategy. The development of vaccinations that mainly target fish TLRs should be the main focus of future studies. Finally, it is essential to comprehend how external factors affect fish TLR expression and activity. Bridging the gap between TLR research and practical use in aquaculture is vital. Understanding the differences in immunology among aquatic species and their mammalian counterparts is an essential area of research in comprehending complex immune responses.
