# Supplementary material for: Unveiling the multifaceted role of toll-like receptors in immunity of aquatic animals: pioneering strategies for disease management
Source: Front Immunol. 2024 Oct 17;15:1378111. doi: 10.3389/fimmu.2024.1378111 (PMC11524855; doi:10.3389/fimmu.2024.1378111)
Supplement: Supplementary file 1 [file DataSheet1.pdf]

Supplementary Material

TABLE S1: Accession number of TLR sequences used for phylogenetic tree

Gene	Organism	Accession number
**TLR1**	*Trachinotus ovatus*	AYM26735.1
	Danio rerio	AAI63271.1
	Ctenopharyngodon idella	ACT68332.1
	Ictalurus punctatus	AEI59662.1
	Labeo rohita	KAI2651395.1
	Larimichthys crocea	AHB51065.1
	Xenopus laevis	XP_018084600.1
	Gallus gallus	BAD67422.1
	Mus musculus	AAG35062.1
	Homo sapiens	AAC34137.1
**TLR2**	*Labeo rohita*	ADQ74644.1
	Danio rerio	AAQ90474.1
	Ctenopharyngodon idella	ACT68333.1
	Trachinotus ovatus	AYM26736.1
	Larimichthys crocea	AHB51066.1
	Xenopus laevis	XP_018089045.1
	Gallus gallus	BAB16113.2
	Mus musculus	AAO21125.1
	Homo sapiens	AAY85647.1
**TLR3**	*Labeo rohita*	KAI2668650.1
	Danio rerio	AAT37633.1
	Ctenopharyngodon idella	ABI64155.1
	Trachinotus ovatus	QCV57274.1
	Larimichthys crocea	ADW79423.1
	Xenopus laevis	XP_018085800.1
	Gallus gallus	APJ35706.1
	Mus musculus	AAK26117.1
	Homo sapiens	AAH94737.1
**TLR4**	*Labeo rohita*	AOM81178.1
	Danio rerio	NM_001131051
	Ctenopharyngodon idella	ACT68334.1
	Gallus gallus	ACR26281.1
	Mus musculus	EDL31078.1
	Xenopus laevis	XP_018088395.2
	Homo sapiens	AAY82270.1
**TLR5**	*Larimichthys crocea*	APJ35912.1
	Danio rerio	AY389449.1
	Trachinotus ovatus	QNM41382.1
	Labeo rohita	XP_019123270.2
	Gallus gallus	KAI2652317.1
	Xenopus tropicalis	AAI21459.1
	Mus musculus	AAF65625.1
	Homo sapiens	ACM69021.1
**TLR6**	*Carassius auratus*	XP_026137066.1
	Cyprinus carpio	XP_042594684.1
	Sinocyclocheilus grahami	XP_016099000.1
	Sinocyclocheilus rhinocerous	XP_016421048.1
	Anabarilius grahami	ROL41152.1

	Astyanax mexicanus	KAG9263780.1
	Gallus gallus	NP_001075178.4
	Mus musculus	AAH55366.1
	Homo sapiens	ABY67114.1
**TLR7**	*Labeo rohita*	KAI2660936.1
	Danio rerio	XP_021334735.1
	Trachinotus ovatus	ANA09010.1
	Xenopus tropicalis	NP_001120883.1
	Gallus gallus	ACR26243.1
	Mus musculus	AAK62676.1
	Homo sapiens	AAZ99026.1
**TLR8**	*Labeo rohita*	KAI2660290.1
	Trachinotus ovatus	ANA09011.1
	Larimichthys crocea	AGO28201.1
	Danio rerio	XP_002665954.4
	Xenopus tropicalis	XP_002933859.2
	Mus musculus	AAK62677.1
	Homo sapiens	AAZ95441.1
**TLR9**	*Labeo rohita*	KAI2661205.1
	Danio rerio	AAI63628.1
	Ctenopharyngodon idella	ADB96920.1
	Trachinotus ovatus	ANA09012.1
	Rana temporaria	XP_040214719.1
	Mus musculus	AAK29625.1
	Homo sapiens	AAZ95517.1
**TLR10**	*Gracilinanus agilis*	XP_044536142.1
	Trichosurus vulpecula	XP_036621291.1
	Pantroglodytes ellioti	AGR83748.1
	Homo sapiens	AAK26744.1
**TLR11**	*Mus musculus*	NP_991388.2
	Sciurus carolinensis	MBZ3888773.1
**TLR12**	*Labeo rohita*	KAI2655291.1
	Xenopus tropicalis	XP_002941870.2
	Mus musculus	AAS37673.1
**TLR13**	*Labeo rohita*	KAI2655342.1
	Danio rerio	XP_002664892.4
	Xenopus tropicalis	XP_002942581.2
	Ctenopharyngodon idella	XP_051722697.1
**TLR14**	*Trachinotus ovatus*	AXL48518.1
	Sebastiscus marmoratus	USC27896.1
	Argyrosomus japonicus	QOS44507.1
	Siniperca chuatsi	QWW30860.1
	Miichthys miiuy	ALJ55572.1
**TLR15**	*Gallus gallus*	ACR26584.1
**TLR16**	*Gallus gallus*	ABQ85926.1
**TLR18**	*Danio rerio*	AAI63840.1
	Labeo rohita	XP_050987524.1
	Ctenopharyngodon idella	AIB55030.1
**TLR19**	*Danio rerio*	NP_001352353.1
	Cyprinus carpio	UFQ05947.1
	Salmo salar	CDH93609.2
**TLR20**	*Triplophysa rosa*	KAI7808871.1
	Megalobrama amblycephala	APT35509.1
	Cyprinus carpio	AHH85805.1
	Ctenopharyngodon idella	AHN49762.1
**TLR21**	*Trachinotus ovatus*	QCI31552.1

	Thunnus albacares	XP_044214792.1
	Danio rerio	CAQ13807.1
	Scophthalmus maximus	AMQ35500.1
	Ctenopharyngodon idella	AGM21642.1
**TLR22**	*Ictalurus punctatus*	XP_017348610.1
	Ctenopharyngodon idella	ADX97523.2
	Danio rerio	NP_001122147.2
	Labeo rohita	AHV90682.1
	Cyprinus carpio	ADR66025.1
**TLR23**	*Takifugu rubripes*	AAW70378.1
	Miichthys miiuy	ALJ55575.1
	Argyrosomus japonicus	QOS44509.1
**TLR25**	*Ictalurus punctatus*	AEI59680.1
	Cyprinus carpio	BAU98397.1
	Ctenopharyngodon idella	AVI26518.1
**TLR26**	*Ictalurus punctatus*	AEI59681.1
	Tachysurus fulvidraco	QLL99516.1
**TLR27**	*Trachinotus ovatus*	AUT31004.1

Table S2:: Abbreviations used in the literature

**Abbreviation**	**Extended Terminology**
TLRs	Toll-like receptors
PAMPs	Pathogen associated molecular patterns
PRRs	Pattern recognition receptors
NOD	Nucleotide-binding oligomerization domain
NLRs	Nucleotide-binding oligomerization domain like receptors
RIG	Retinoic acid-inducible gene-I
RLRs	Retinoic acid-inducible gene-I-like receptors
LRR	Leucine-rich repeat regions
SMART	Simple Modular Architecture Research Tool
NTD	N-terminal domain
TIR	Toll/Interleukin-1 receptor domain
CTD	Cytoplasmic terminal Domain
TMD	Transmembrane domain
ECD	Extracellular domain
MD2	Myeloid Differentiation 2
MyD88	Myeloid Differentiation Primary Response 88
TRIF	Toll/interleukin-1 receptor (TIR) domain-containing adapter-inducing interferon-β
AMPs	Antimicrobial Peptides
CpG	Cytosine phosphate Guanine
LPS	Lipopolysaccharides
PGN	Peptidoglycan
LTA	Lipoteichoic acid
ProPO	Prophenoloxidase
NF-kβ	Nuclear factor Kappa beta
poly I: C	Polyinosinic: polycytidylic acid
TNF-α	Tumor necrotic factor α
TICAM1	Toll-Interleukin 1 Receptor (TIR) Domain Containing Adaptor Molecule 1
MALP-2	Macrophage-Activating Lipopeptide-2
LBP	Lipopolysaccharides Binding Protein

CpG-ODN	Cytosine-phosphate-Guanine Oligodeoxynucleotide
